# Maternal Mental Health Status and Approaches for Accessing Antenatal Care Information During the COVID-19 Epidemic in China: Cross-Sectional Study

**DOI:** 10.2196/18722

**Published:** 2021-01-18

**Authors:** Hong Jiang, Longmei Jin, Xu Qian, Xu Xiong, Xuena La, Weiyi Chen, Xiaoguang Yang, Fengyun Yang, Xinwen Zhang, Nazhakaiti Abudukelimu, Xingying Li, Zhenyu Xie, Xiaoling Zhu, Xiaohua Zhang, Lifeng Zhang, Li Wang, Lingling Li, Mu Li

**Affiliations:** 1 School of Public Health Fudan University Shanghai China; 2 Key Lab of Health Technology Assessment (National Health Commission) Fudan University Shanghai China; 3 Global Health Institute Fudan University Shanghai China; 4 Minhang Branch, School of Public Health Fudan University Shanghai China; 5 Minhang Maternal and Child Health Care Hospital Shanghai China; 6 School of Public Health and Tropical Medicine Tulane University New Orleans, LA United States; 7 Jiading Maternal and Child Health Care Hospital Shanghai China; 8 The Fourth People’s Hospital of Shaanxi Province Xi’an China; 9 Pudong New District Maternal and Child Health Care Hospital Shanghai China; 10 Leping Maternal and Child Health Care Hospital Leping China; 11 Changzhou Maternal and Child Health Care Hospital Changzhou China; 12 Changzheng Hospital Second Military Medical University Shanghai China; 13 School of Public Health The University of Sydney Sydney Australia; 14 China Studies Centre The University of Sydney Sydney Australia

**Keywords:** COVID-19, mental health, perceived stress, anxiety, depression, antenatal care information, social media platform, pregnancy, women

## Abstract

**Background:**

China was the first country in the world to experience a large-scale COVID-19 outbreak. The rapid spread of the disease and enforcement of public health measures has caused distress among vulnerable populations such as pregnant women. With a limited understanding of the novel, emerging infectious disease, pregnant women have sought ways to access timely and trusted health care information. The mental health status of pregnant women during this public health emergency, as well as how they responded to the situation and where and how they obtained antenatal care information, remain to be understood.

**Objective:**

This study aimed to evaluate the mental health status of pregnant women during the COVID-19 epidemic in China by measuring their perceived stress, anxiety, and depression levels; explore the approaches used by them to access antenatal health care information; and determine their associations with maternal mental health status.

**Methods:**

We conducted a web-based, cross-sectional survey to assess the mental health status of Chinese pregnant women by using the validated, Chinese version of Perceived Stress Scale, Self-Rating Anxiety Scale, and Edinburgh Depression Scale. We also collected information on the various approaches these women used to access antenatal care information during the early stage of the COVID-19 epidemic, from February 5 to 28, 2020.

**Results:**

A total of 1873 pregnant women from 22 provinces or regions of China participated in the survey. The prevalence of perceived stress, anxiety, and depression among these participants was 89.1% (1668/1873; 95% CI 87.6%, 90.4%), 18.1% (339/1873; 95% CI 16.4%, 19.9%), and 45.9% (859/1873; 95% CI 43.6%, 48.1%), respectively. Hospitals’ official accounts on the Chinese social media platforms WeChat and Weibo were the most popular channels among these pregnant women to obtain antenatal care information during the COVID-19 outbreak. Access to antenatal care information via the hospitals’ official social media accounts was found to be associated with a significantly lower risk of perceived stress (adjusted odds ratio [aOR] 0.46, 95% CI 0.30-0.72; *P=*.001), anxiety (aOR 0.53, 95% CI 0.41-0.68; *P*<.001), and depression (aOR 0.73, 95% CI 0.59-0.91; *P*=.005). Access to health care information via hospital hotlines or SMS was found to be significantly associated with a lower risk of anxiety only (OR 0.77, 95% CI 0.60-0.98; *P*=.04).

**Conclusions:**

During the COVID-19 outbreak in China, pregnant women experienced high levels of perceived stress, anxiety, and depression. During such public health emergencies, mental health care services should be strengthened to reassure and support pregnant women. Specific information targeted at pregnant women, including information on how to cope in an emergency or major disease outbreak, developed and disseminated by health care institutions via social media platforms could be an effective way to mitigate mental health challenges and ensure epidemic preparedness and response in the future.

## Introduction

COVID-19 first emerged in Wuhan city in China in December 2019 [1,2]. Soon after, the Chinese government initiated the level-one (ie, the highest level) response to major public health emergencies [3], and the World Health Organization declared the COVID-19 outbreak as a Public Health Emergency of International Concern on January 30, 2020. By February 29, 2020, the number of confirmed COVID-19 cases in China reached over 79,000, with 2800 deaths reported and 20% of the cases classified as severe [4]. March onward, the COVID-19 outbreak spread to almost all nations worldwide and evolved into a full-blown pandemic [5]. By the end of August 2020, more than 28 million confirmed COVID-19 cases and over 900,000 deaths had been reported worldwide [6]. The Chinese government implemented timely measures, including city lockdown, self-quarantine, and social distancing across the nation to contain the transmission of COVID-19. People were advised to stay at home and avoid going to public places and social gatherings [7]. The rapid spread of the epidemic and the consequent enforcement of public health measures had unavoidably raised fear and panic reaction among the public [8-10].

Pregnant women and their unborn children are especially vulnerable to major epidemics or natural disasters [11,12]. During the early stage of the COVID-19 epidemic in China, with limited information and understanding about the new emerging infectious disease, pregnant women looked for different ways to access timely and trusted information regarding the causes, possible routes of transmission, prevention, and self-protection approaches to protect themselves and their unborn children [13]. Given the strict public health emergency measures such as travel restriction and social distancing, pregnant women had been isolated from their professional and social support, including hospitals that provided routine antenatal care services. Such interrupted contact with health institutions could exacerbate uncertainty among these women about service provision and their own health status. Routine maternal health services have often been affected during major infectious disease outbreaks in the past. For example, during the 2003 SARS outbreak, all antenatal services in Hong Kong, China, which were considered nonessential were suspended, and women were discharged as early as possible after childbirth [14]. Since the announcement of the level-one response to major public health emergencies was made in China in late January 2020 for COVID-19, some hospitals focused on containing the spread of COVID-19, and obstetric-related services were suspended as a result [15]. Furthermore, procedures of accessing maternal service were modified; for instance, most hospitals required prior online registration and some hospitals limited the number of family members accompanying the pregnant woman to her antenatal clinic appointment to a maximum of one person[16]. A previous review has shown that during infectious disease outbreaks, pregnant women are likely to experience psychological distress; they are often concerned about the wellbeing of their fetus, when faced with the decision of whether to comply with the recommended prevention and treatment guidelines with their effectiveness and potential side effects yet to be understood, and the disrupted routines of maternal health services [16]. Thus, timely access to information about antenatal care services and education about public health challenges, as well as knowledge of self-protection from trusted sources, could play important roles in reassuring and supporting pregnant women during a public health emergency.

Like the popularity of Facebook and WhatsApp in many countries, a number of homegrown social media platforms such as WeChat and Weibo have penetrated the daily lives of Chinese people. WeChat is now the most popular mobile phone app in China with over 654 million users as of 2019, followed by Weibo with nearly 360 million users as of 2019 [17]. An increasing number of private enterprises, government organizations, and hospitals have established WeChat or Weibo official accounts (WOAs) to disseminate service information and promote interaction with end users. WeChat and Weibo are also the two most popular social media platforms used by hospitals to inform the public about the services they offered and provide health education and counselling free of charge.

China was the first county in the world to experience the COVID-19 outbreak. Understanding how the epidemic and public health measures, including restricted mobility, affected maternal mental health and whether the ways of accessing antenatal care information affected the mental wellbeing of pregnant women would provide important scientific information to develop effective support strategies and preparatory mechanisms for future global public health emergencies. Therefore, the aims of this study were to (1) determine the mental health status of pregnant women during the early stage of the COVID-19 epidemic in China, by measuring their perceived stress, anxiety, and depression levels; (2) explore the various approaches used by them to access antenatal care information; and (3) determine their associations with the maternal mental health status.

## Methods

### Study Design

We conducted a web-based, cross-sectional survey from February 5 to 28, 2020, during the peak of the COVID-19 epidemic in China. The questionnaire was designed using the online survey tool Wenjuanxing platform [18]. The contents of the survey gathered participants’ demographic characteristics and evaluated their COVID-19 self-protection behaviors, knowledge of antenatal COVID-19–related care, mental health status, and channels of obtaining antenatal care information. The study was approved by the Institutional Review Board of School of Public Health, Fudan University, Shanghai, China (IRB#2020-02-0803).

### Data Collection

We used direct online and snowball recruitment methods through our research collaboration network in an attempt to achieve higher participation from different regions of China. We sent the quick response (QR) code or the link to the survey questionnaire to the heads of maternal and child health (MCH) departments of MCH hospitals and comprehensive hospitals; these included 2 district MCH hospitals in Shanghai and 1 municipal MCH hospital in Changzhou, Jiangsu Province; 1 county MCH hospital in Leping, Jiangxi Province; and 1 municipal comprehensive hospital in Xi’an, Shaanxi Province. These department heads sent the electronic QR code or the survey URL to the staff of their respective departments and posted the recruitment details on the walls of the antenatal clinics. Pregnant women who visited the antenatal clinics in these facilities during the survey period were invited to participate in the survey; they were required to either scan the QR code or access the URL via a mobile phone or other digital devices. On the first page of the survey, a brief introduction to the survey, including the purpose and contents of the web-based questionnaire and the estimated time to complete the survey, was provided. At this point, participants could either choose to continue to answer the questionnaire or close the page to exit the survey. Participants were also encouraged to share the survey QR code or URL with other pregnant women in their network. Upon clicking the “submit” button at survey completion, key antenatal care knowledge relevant to COVID-19 (which were also the answers to the questions) would be displayed on the screen.

### Survey Design

Participants’ sociodemographic and obstetric information, including age, education level, status of employment, parity, gestational age, and pregnancy complication, were collected. The survey comprised 10 questions regarding personal self-protection behaviors to prevent COVID-19 and knowledge of antenatal care required during the COVID-19 epidemic, based on the joint Chinese professional societies’ guidelines [19]. The 4 behavior questions, including 2 multiple choice questions, were about (1) avoiding stepping out of the home, (2) wearing a face mask in public places, (3) practicing hand sanitization, and (4) practicing other protection behaviors such as avoiding using the public transport and avoiding contact with wild animals and consumption of wild animal meat. The 6 antenatal care questions, including 3 multiple choice questions, evaluate participant’s knowledge of (1) whether the antenatal health visit should be cancelled, (2) whether pregnant women could undergo a computed tomography scan, (3) symptoms urging pregnant women to visit the hospital, (4) where to seek care in case of fever, (5) should newborn babies be quarantined if the mother is diagnosed with COVID-19, and (6) whether breastfeeding is recommended if the mother has COVID-19 (see Multimedia Appendix 1). Each question was assigned an individual score. The total score of self-protection behaviors was 4 and that of antenatal care knowledge was 6. Survey participants were categorized into the following 2 groups if their total scores were above the median score: better self-protection behavior group and higher antenatal care knowledge group.

### Mental Well-Being Assessment

#### Perceived Stress

We used the validated Chinese Perceived Stress Scale (CPSS) to assess perceived stress among pregnant women [20]. The CPSS is a 14-item, self-reported questionnaire with 7 positive and 7 negative items measuring the degree to which individuals appraise situations in their lives as stressful [21,22]. Each item is scored on a 5-point Likert scale ranging from 1 (“never”) to 5 (“very often”) [22]. The total score of this scale is computed by summing the scores of all positive items and reverse-scoring all negative items. The cutoff point for CPSS for the Chinese population is 25. Therefore, participants who scored higher than 25 were considered to be experiencing perceived stress [22-24].

#### Anxiety

The validated, 20-item Chinese version of the Self-Rating Anxiety Scale (SAS) was used to measure participants’ anxiety symptoms [25]. Each item on the SAS is scored using a 4-point Likert scale, with scores ranging from 1 (“a little of the time”) to 4 (“most of the time”). Items 5, 9, 13, 17, and 19 are negatively keyed. The total score is computed by multiplying the sum of the scores of positive items and the reverse-scores of negative items by 1.25. The total score of SAS ranges from 20 to 80 [26]. Participants scoring 50 and above were considered to be experiencing anxiety [27,28].

#### Depression

The 10-item Chinese version of the Edinburgh Depression Scale (EDS) was used to evaluate maternal depression [29]. Each item was scored on a 4-point Likert scale, with scores ranging from 0 to 3. The total score ranges from 0 to 30, and the cut-off scores range from 9 to 13. For this study, we considered a cut-off score of 9. Participants with a score of 10 and above were considered to be experiencing depression [30-32].

### Accessing Antenatal Care Information

The question on how participants accessed antenatal care information during the COVID-19 epidemic was in a multiple-choice format: hospitals’ hotline, mobile phone SMS, hospitals’ official WeChat accounts, hospitals’ official Weibo accounts, WeChat or Weibo moments (an information sharing forum) posted by friends and family members, and digital message or verbal advice shared by friends and family members. Participants who selected either the hospitals’ official WeChat or Weibo accounts were categorized as accessing information via hospitals’ official accounts on social media platforms. Those who selected WeChat or Weibo moments from friends and family members or other means involving friends and family members were categorized as accessing information via friends and family members.

Accessing antenatal care information via hotlines or SMS represents accessing antenatal care information from a reliable source and using a traditional approach. Accessing health care information via hospital official accounts on social media represents accessing antenatal care information via a reliable source and using a social media approach. Accessing health care information via friends or family members represents accessing information from nonprofessional sources. Analysis of these 3 different approaches will help clarify the roles of both the information sources and access channels.

### Quality Control

For the purpose of quality control of the web-based survey, we incorporated 1 question after the CPSS scale: “The purpose of this question is to verify if a participant has answered the question carefully. Please select ‘always’ as you are told.” If a participant failed to answer the question as required, their questionnaire response would be regarded as invalid. Among the 2186 pregnant women who participated in the survey, 1873 returned a valid questionnaire.

### Data Analysis

We evaluated the mean (SD) values for normally distributed continuous variables and median (P25-P75) values for non-normally distributed variables. Categorical variables were described in proportions. Chi-square test was used to analyze the categorical variables, and multiple binary logistic regression analyses were performed to examine the associations between different approaches of accessing antenatal care information and stress, anxiety, and depression after adjusting for social economic factors such as age; education; employment status; living in urban, suburban, or rural areas; province or region of current residence; obstetric conditions (eg, parity, trimester, and presence of complications); score of COVID-19 prevention self-protection behaviors; and score of COVID-19–related antenatal care knowledge. The significance level was set at *P*<.05, and data were analyzed using SPSS software for Windows (version 17.0; SPSS Inc.) and R Statistical Software (version 3.6.3).

## Results

The mean age of the 1873 participants was 29 (SD 4.10) years. The distribution of women’s current residence covered 22 provinces or regions of China, with the majority of participants based in Shanghai (1415/1873, 75.5%) and a small proportion based in Hubei Province (23/1873, 1.2%)—the most severely affected area due to the COVID-19 outbreak. The majority of women had completed college and above level of education, were employed, and primiparous. The number of pregnant women in their first, second, and third trimesters was almost evenly distributed. The median score of COVID-19 self-protection behavior was 3, with individual scores ranging from 1 to 4. The median score of antenatal care knowledge was 4, with individual scores ranging from 0 to 6 (Table 1).

**Table 1:** Characteristics of pregnant women and their approaches of accessing antenatal care information during the COVID-19 outbreak in China (N=1873).

Characteristic		Accessing antenatal care information via hospital hotlines or SMS					Accessing health care information via hospital official accounts on social media platforms					Accessing health care information via friends or family members			
		No, n (%)^a^	Yes, n (%)	Chi-square (*df*)	*P* value^b^	No, n (%)		Yes, n (%)	Chi-square (*df*)	*P* value^b^	No, n (%)		Yes, n (%)	Chi-square (*df*)	*P* value^b^
Overall		988 (52.7)	885 (47.3)	—^c^	—	457 (24.4%)		1416 (75.6)	—	—	1127 (60.2)		746 (39.8)	—	—
**Age (years)^d^**				0.9 (1)	.34				4.8 (1)	.03				4.6 (1)	.03
	<29	373 (51.4)	353 (48.6)			197 (27.1)		529 (72.9)			459 (63.2)		267 (36.8)		
	≥29	615 (53.6)	532 (46.4)			260 (22.7)		887 (77.3)			668 (58.2)		479 (41.8)		
**Education level**				10.7 (2)	.005				51.8 (2)	<.001				7.8 (2)	.02
	Junior high or lower	103 (46)	121 (54)			88 (39.3)		136 (60.7)			143 (63.8)		81 (36.2)		
	Senior high	130 (46.9)	147 (53.1)			92 (33.2)		185 (66.8)			184 (66.4)		93 (33.6)		
	College or higher	755 (55)	617 (45)			277 (20.2)		1095 (79.8)			800 (58.3)		572 (41.7)		
**Employment status**				1.1 (1)	.30				8.8 (1)	.003				2.3 (1)	.13
	Unemployed	140 (55.8)	111 (44.2)			80 (31.9)		171 (68.1)			162 (64.5)		89 (35.5)		
	Employed	848 (52.3)	774 (47.7)			377 (23.2)		1245 (76.8)			965 (59.5)		657 (40.5)		
**Parity**				17.9 (1)	<.001				1.5 (1)	.23				3.1 (1)	.08
	Primiparous	685 (56.3)	531 (43.7)			286 (23.5)		930 (76.5)			714 (58.7)		502 (41.3)		
	Multiparous	303 (46.1)	354 (53.9)			171 (26)		486 (74)			413 (62.9)		244 (37.1)		
**Trimester**				21.1 (2)	<.001				18.6 (2)	<.001				3.2 (2)	.20
	1st	271 (45.3)	327 (54.7)			112 (18.7)		486 (81.3)			348 (58.2)		250 (41.8)		
	2nd	384 (54.6)	319 (45.4)			204 (29)		499 (71)			441 (62.7)		262 (37.3)		
	3rd	333 (58.2)	239 (41.8)			141 (24.7)		431 (75.3)			338 (59.1)		234 (40.9)		
**Living area**				0.4 (2)	.83				36.0 (2)	<.001				4.8 (2)	.09
	Urban	699 (52.5)	632 (47.5)			286 (21.5)		1045 (78.5)			781 (58.7)		550 (41.3)		
	Suburban	174 (52.4)	158 (47.6)			86 (25.9)		246 (74.1)			208 (62.7)		124 (37.3)		
	Rural	115 (54.8)	95 (45.2)			85 (40.5)		125 (59.5)			138 (65.7)		72 (34.3)		
**Current residence**				21.8 (1)	<.001				7.1 (1)	.008				0.0 (1)	.96
	Non-Shanghai	285 (62.2)	173 (37.8)			133 (29)		325 (71)			276 (60.3)		182 (39.7)		
	Shanghai	703 (49.7)	712 (50.3)			324 (22.9)		1091 (77.1)			851 (60.1)		564 (39.9)		
**Pregnancy complications**				0.4 (1)	.53				0.8 (1)	.38				0.1 (1)	.75
	No	737 (53.2)	649 (46.8)			331 (23.9)		1055 (76.1)			831 (60)		555 (40)		
	Yes	251 (51.5)	236 (48.5)			126 (25.9)		361 (74.1)			296 (60.8)		191 (39.2)		
**Median score of COVID-19 prevention self-protection behavior**				10.8 (1)	.001				4.3 (1)	.04				9.8 (1)	.002
	Low	784 (54.9)	645 (45.1)			365 (25.5)		1064 (74.5)			888 (62.1)		541 (37.9)		
	High	204 (45.9)	240 (54.1)			92 (20.7)		352 (79.3)			239 (53.8)		205 (46.2)		
**Median score of COVID-19 antenatal care knowledge**				2.0 (1)	.16				7.3 (1)	.007				3.5 (1)	.06
	Low	722 (51.8)	672 (48.2)			362 (26)		1032 (74)			856 (61.4)		538 (38.6)		
	High	266 (55.5)	213 (44.5)			95 (19.8)		384 (80.2)			271 (56.6)		208 (43.4)		

^a^Percentages reported parenthetically are based on the sum of absolute values of each subcategory (or row) listed under “Characteristic.”

^b^Chi-square test.

^c^Data not available.

^d^The mean age of 29 years was used to categorize participants into the 2 groups: ≥29 and <29 years.

The mean score of perceived stress was 35.21 (SD 7.58), as measured by CPSS. The median scores of anxiety and depression as measured by SAS and EDS were 42.50 (IQR 37.50-47.50) and 9.00 (IQR 7.00, 11.00), respectively. The prevalence of perceived stress, anxiety, and depression symptoms among the participants was reported to be 89.1% (1668/1873; 95% CI 87.6%, 90.4%), 18.1% (339/1873; 95% CI 16.4%, 19.9%), and 45.9% (859/1873; 95% CI 43.6%, 48.1%), respectively. Univariate analysis showed that pregnant women who were employed, lived in Shanghai, had completed higher education, and had scored higher for COVID-19 prevention knowledge and self-protection behaviors showed a negative association for experiencing at least one form of the 3 mental health disorders assessed. On the other hand, pregnant women who were multiparous, resided in rural areas, and had pregnancy complications were more likely to experience at least one form of the 3 mental health disorders assessed, compared to those who were primiparous, lived in urban areas, and did not have pregnancy complications (Table 2).

**Table 2:** Characteristics of pregnant women and their perceived stress, anxiety, and depression levels (N=1873).

Characteristic		Perceived stress				Anxiety				Depression			
		No, n (%)^a^	Yes, n (%)	Chi-square (*df*)	*P* value^b^	No, n (%)	Yes, n (%)	Chi-square (*df*)	*P* value^b^	No, n (%)	Yes, n (%)	Chi-square (*df*)	*P* value^b^
Overall		205 (10.9)	1668 (89.1)	—^c^	—	1534 (81.9)	339 (18.1)	—	—	1014 (54.1)	859 (45.9)	—	—
**Age** **(years)^d^**				2.5 (1)	.11			1.4 (1)	.24			0.1 (1)	.70
	<29	69 (9.5)	657 (90.5)			585 (80.6)	141 (19.4)			389 (53.6)	337 (46.4)		
	≥29	136 (11.9)	1011 (88.1)			949 (82.7)	198 (17.3)			625 (54.5)	522 (45.5)		
**Education**				17.1 (2)	<.001			28.9 (2)	<.001			22.5 (2)	<.001
	Junior high or below	10 (4.5)	214 (95.5)			159 (71)	65 (29)			102 (45.5)	122 (54.5)		
	Senior high	21 (7.6)	256 (92.4)			214 (77.3)	63 (22.7)			124 (44.8)	153 (55.2)		
	College or above	174 (12.7)	1198 (87.3)			1161 (84.6)	211 (15.4)			788 (57.4)	584 (42.6)		
**Employment status**				4.2 (1)	.04			2.3 (1)	.13			3.1 (1)	.08
	Unemployed	18 (7.2)	233 (92.8)			197 (78.5)	54 (21.5)			123 (49)	128 (51)		
	Employed	187 (11.5)	1435 (88.5)			1337 (82.4)	285 (17.6)			891 (54.9)	731 (45.1)		
**Parity**				0.6 (1)	.45			2.3 (1)	.13			5.8 (1)	.02
	Primiparous	138 (11.3)	1078 (88.7)			1008 (82.9)	208 (17.1)			683 (56.2)	533 (43.8)		
	Multiparous	67 (10.2)	590 (89.8)			526 (80.1)	131 (19.9)			331 (50.4)	326 (49.6)		
**Trimester**				2.9 (2)	.23			1.2 (2)	.56			0.5 (2)	.78
	1st	75 (12.5)	523 (87.5)			490 (81.9)	108 (18.1)			330 (55.2)	268 (44.8)		
	2nd	76 (10.8)	627 (89.2)			583 (82.9)	120 (17.1)			380 (54.1)	323 (45.9)		
	3rd	54 (9.4)	518 (90.6)			461 (80.6)	111 (19.4)			304 (53.1)	268 (46.9)		
**Living area**				9.3 (2)	.01			8.2 (2)	.02			9.8 (2)	.008
	Urban	157 (11.8)	1174 (88.2)			1103 (82.9)	228 (17.1)			743 (55.8)	588 (44.2)		
	Suburban	38 (11.4)	294 (88.6)			274 (82.5)	58 (17.5)			178 (53.6)	154 (46.4)		
	Rural	10 (4.8)	200 (95.2)			157 (74.8)	53 (25.2)			93 (44.3)	117 (55.7)		
**Current residence**				17.3 (1)	<.001			7.9 (1)	.005			5.1 (1)	.02
	Non-Shanghai	26 (5.7)	432 (94.3)			355 (77.5)	103 (22.5)			227 (49.6)	231 (50.4)		
	Shanghai	179 (12.7)	1236 (87.3)			1179 (83.3)	236 (16.7)			787 (55.6)	628 (44.4)		
**Pregnancy complications**				2.5 (1)	.12			5.3 (1)	.02			1.8 (1)	.18
	No	161 (11.6)	1225 (88.4)			1152 (83.1)	234 (16.9)			763 (55.1)	623 (44.9)		
	Yes	44 (9)	443 (91)			382 (78.4)	105 (21.6)			251 (51.5)	236 (48.5)		
**Score of COVID-19 prevention self-protection behaviors**				1.2 (1)	.27			0.6 (1)	.45			0.3 (1)	.61
	Low	150 (10.5)	1279 (89.5)			1165 (81.5)	264 (18.5)			769 (53.8)	660 (46.2)		
	High	55 (12.4)	389 (87.6)			369 (83.1)	75 (16.9)			245 (55.2)	199 (44.8)		
**Score of COVID-19 antenatal care knowledge**				11.0 (1)	.001			7.3 (1)	.007			3.9 (1)	.047
	Low	133 (9.5)	1261 (90.5)			1122 (80.5)	272 (19.5)			736 (52.8)	658 (47.2)		
	High	72 (15)	407 (85)			412 (86)	67 (14)			278 (58)	201 (42)		
**Access to antenatal care information via hotlines or SMS^b^**				1.1 (1)	.29			2.5 (1)	.11			0.3 (1)	.59
	No	101 (10.2)	887 (89.8)			796 (80.6)	192 (19.4)			529 (53.5)	459 (46.5)		
	Yes	104 (11.8)	781 (88.2)			738 (83.4)	147 (16.6)			485 (54.8)	400 (45.2)		
**Access to antenatal care information via hospitals’ official accounts on social media platforms**				18.6 (1)	<.001			33.3 (1)	<.001			13.8 (1)	<.001
		25 (5.5)	432 (94.5)			333 (72.9)	124 (27.1)			213 (46.6)	244 (53.4)		
		180 (12.7)	1236 (87.3)			1201 (84.8)	215 (15.2)			801 (56.6)	615 (43.4)		
**Access to antenatal care information from friends or family members**				2.1 (1)	.15			0.1 (1)	.81			6.0 (1)	.01
		133 (11.8)	994 (88.2)			925 (82.1)	202 (17.9)			636 (56.4)	491 (43.6)		
	Yes	72 (9.7)	674 (90.3)			609 (81.6)	137 (18.4)			378 (50.7)	368 (49.3)		

^a^Percentages reported parenthetically are based on the sum of absolute values of each subcategory (or row) listed under “Characteristic.”

^b^Chi-square test.

^c^Data not available.

^d^The mean age of 29 years was used to categorize participants into the 2 groups: ≥29 and <29 years.

**Figure 1:**
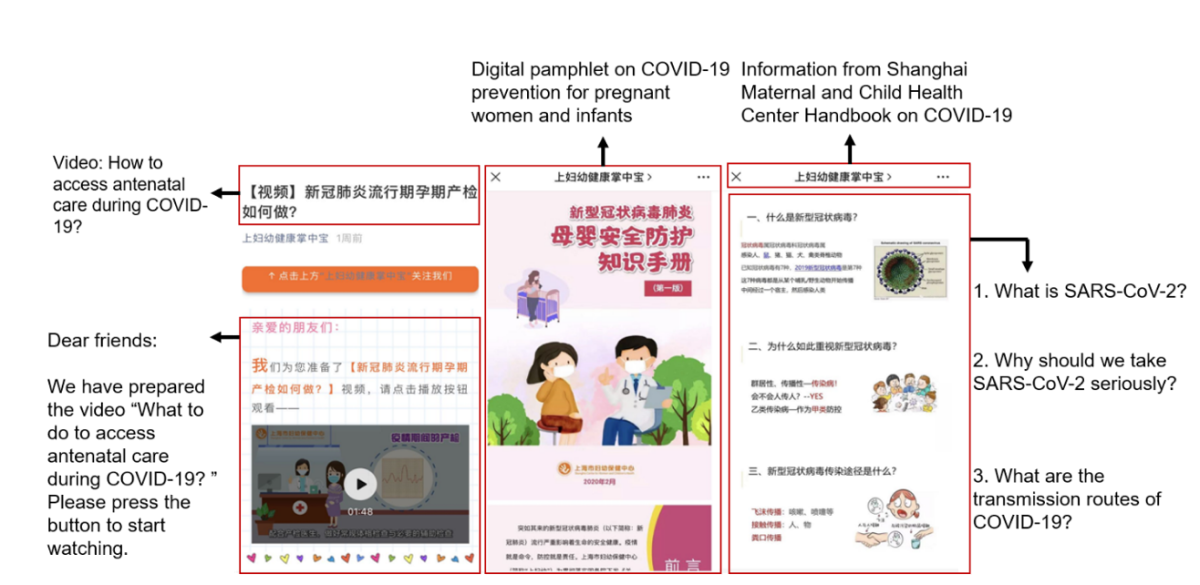
Examples of antenatal care information provided via a health institution’s official WeChat account. Screenshots of antenatal care information shared via the official WeChat account of Shanghai Maternal and Child Health Center, China, introducing when, where, and how should pregnant women receive antenatal care and COVID-19 prevention strategies for pregnant women and infants.

In all, 75.6% (1416/1873) of the participants reported having ever accessed antenatal health care information via hospitals’ WOAs, and 63.2% (1185/1873) of the participants also selected WOAs as the most preferred way to access antenatal care information (Figure 1). Moreover, 47.3% (885/1873) of the participants reported they accessed antenatal care information via hospital hotlines or SMS, and 39.8% (746/1873) accessed this information via friends or family members. Results of the chi-square test showed that women who were relatively older, had received higher education, were employed, in their 1st or 3rd trimester, residing in urban areas, and living in Shanghai area were more likely to access information via hospitals’ WOAs. All 3 different approaches of accessing antenatal care information were positively associated with a higher score of COVID-19 self-protection behaviors. Access to information via WOAs was positively associated with a higher score of COVID-19 antenatal care knowledge (Table 1).

**Figure 2:**
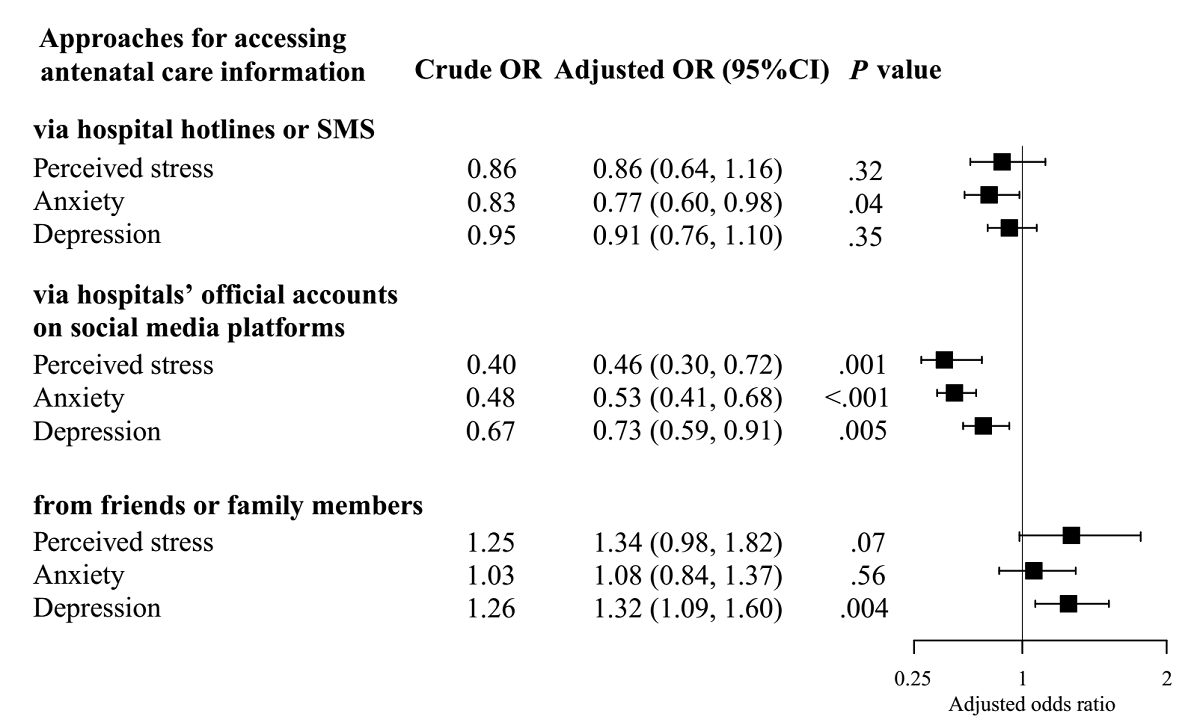
Approaches used by Chinese pregnant women to access antenatal care information and mental health disorders experienced by them during the COVID-19 epidemic (N=1873). Multiple binary logistic regression analysis controlled for age, education level, employment status, living area, current residence, gestational stage (trimester), parity, pregnancy complications, and scores of COVID-19 self-protection behaviors and antenatal care knowledge. OR: odds ratio.

Results of the multiple binary logistic regression analyses showed that participants who accessed antenatal care information via hospitals’ WOAs were significantly associated with lower risk of perceived stress (odds ratio [OR] 0.46, 95% CI 0.30-0.72; *P*=.001), anxiety (OR 0.53, 95% CI 0.41-0.68; *P*<.001) and depression (OR 0.73, 95% CI 0.59-0.91; *P*=.005; see the table in Multimedia Appendix 2). Furthermore, participants who accessed health care information via hospital hotlines or SMS were found to be significantly associated with a lower risk of anxiety only (OR 0.77, 95% CI 0.60-0.98; *P*=.04; see the table in Multimedia Appendix 3). Participants who accessed antenatal care information via friends and family members were found to be significantly associated with a higher risk of experiencing depression symptoms (OR 1.32, 95% CI 1.09-1.60; *P*=.004; see Figure 2 and the table in Multimedia Appendix 4).

Multiple binary logistic regression analysis also showed that factors such as education level of college and above, current residence in Shanghai, and a higher score of COVID-19 antenatal care knowledge were negatively associated with at least one of the mental health disorders assessed in this study, compared with other factors such as a lower education level, current residence outside Shanghai, and a lower score of COVID-19 antenatal care knowledge. Moreover, pregnancy complications were found to be significantly associated with an increased risk of anxiety (Multimedia Appendices 2-4).

## Discussion

### Principal Findings

Our study provides a snapshot of the mental health status of pregnant women during the early stage of the COVID-19 epidemic in China. The prevalence of perceived stress, anxiety, and depression among the pregnant women was high, reported among 89.1%, 18.1%, and 45.9% of the participants, respectively. In addition, our survey results showed that the most popular way to access antenatal care information was via hospitals’ official accounts on social media platforms such as WeChat and Weibo; women who accessed antenatal care information using this approach had a significantly lower risk of perceived stress, anxiety, and depression, after controlling for social economic factors such as age, education, and employment status; obstetric conditions such as gestational stage (trimester) and pregnancy complications; and scores of COVID-19 self-protection behaviors and antenatal care knowledge.

The proportion of women experiencing perceived stress, anxiety, and depression was higher in this study sample, that is, during the COVID-19 epidemic, than that reported in previous Chinese studies in the context of no public health emergency, using the same measurements. For instance, in previous studies, perceived stress was reported at 73.3% [33], anxiety at 11.3% [34,35], and depression at 17.6%-25.4% [32,36]. The higher prevalence of maternal mental health disorders in our study sample was consistent with the findings of other studies conducted during the COVID-19 outbreak [37-40]. The higher risk of maternal mental health disorders might be related to the concerns pregnant women have regarding the COVID-19 pandemic and its impact on the economic and individual social circumstances, such as prolonged stay-at-home, increased exposure to news or rumors, isolation from their social support network and health care facilities, limited access to self-protection resources (eg, face mask and sanitizers), and worry about contracting COVID-19 and mother-to-fetus transmission [16,37]. These concerns and fears could perpetuate stress and anxiety among pregnant women and contribute to increased rates of mental health disorders [38]. Furthermore, compared with the prevalence of depression symptoms (29.6%) in a Chinese study on pregnant women in late January 2020 (ie, at the beginning of the COVID-19 epidemic in China), the prevalence of depression symptoms in our study was much higher (45.9%) [39]. This difference might be due to the time difference between the two studies. Our study was carried out in February 2020, during the peak of the COVID-19 epidemic in China when the number of daily new confirmed cases reached new records and major public health disease control and prevention measures were universally implemented across the country. Although these measures were necessary to contain the spread of the infection, they could also aggravate negative psychological effects among pregnant women [40,41]. There is solid evidence for the link among maternal stress, anxiety, and depression, as well as a number of pregnancy complications and adverse pregnancy and birth outcomes (eg, susceptibility to infection, low birth weight, preterm birth, and impaired cognitive development of the child) [42-44]. The high prevalence of mental health disorders among pregnant women found in this study suggests the needs for timely intervention and preparedness for maternal mental health care in a public health emergency context. Further prospective studies are required to understand the effects of maternal mental health disorders during COVID-19 on pregnancy outcomes and the well-being of the child.

Our findings revealed that access to antenatal care information via hospitals’ WOAs was significantly associated with a lower risk of all 3 measured mental health disorders (ie, perceived stress, anxiety, and depression). Obtaining health care information via hospital hotlines or SMS was found to be significantly associated with a lower risk of anxiety only. Nevertheless, both these approaches highlighted the importance of the source of antenatal care information. Hospitals usually are regarded as a reliable and credible source for obtaining health information. The antenatal care knowledge provided by hospitals during the COVID-19 pandemic is likely to enhance the confidence and self-efficacy of pregnant women to deal with the situation. Furthermore, the feeling of being connected with the hospitals and health care providers via hospital WOAs as well as hotline and SMS during the COVID-19 pandemic is likely to provide reassurance and comfort to pregnant women, which may help mitigate distress. However, unlike the hotline, WOAs allow pregnant women to access the information they need at any time as per their convenience. Moreover, compared with SMS, WOAs offer these women the flexibility to obtain more comprehensive information that meets their needs. WOAs can provide substantial information in various ways, including tips, articles, images, and links to videos. These might be some of the possible reasons why access to information via hospitals’ WOAs was found to be associated with a higher reduction in mental health disorders among pregnant women compared with access to information via hotlines and SMS.

Surprisingly, we found that pregnant women who obtained antenatal care information via friends and family members were associated with a higher risk of depression. This finding could be partially explained by the fact that women experiencing depression symptoms are more likely to turn to their friends and family for help or that they received more concerns from their friends or family members. Nevertheless, this observation suggested the need for actively disseminating maternal health service information and involving family members in antenatal health education. Being able to access health information from a reliable and credible source has always been a primary service need of new and expecting mothers [45]. The findings of this study highlight the importance of both the trustworthiness of the source and the channels of information acquisition. The findings also suggest that during a public health emergency, reliable information provided by health professional institutions via social media platforms is a feasible and potentially effective way to deliver health care information and services to pregnant women. The findings also signify the importance and necessity of communicating authoritative information by health service providers via social media platforms as one component of epidemic preparedness and response in the future.

The Chinese government and many professional societies have responded swiftly to emergency maternal health interventions during the COVID-19 epidemic. Several professional organizations jointly issued management guidelines for pregnancy during COVID-19 on January 31, 2020 [19]. The National Health Commission of China released a province-wise list of designated hospitals for suspected or confirmed COVID-19 cases among pregnant women on February 12, 2020 [46]. Following these, many MCH institutions have posted service information and guidance for antenatal care on WOAs (Figure 1) [47,48]. However, training and capacity building is urgently needed for translating evidence-based information to health education materials in a timely manner such that it is comprehensible by the public. As an increasing number of health institutions choose to provide telemedicine services such as counselling and consultation, the quality of these remote-based services needs to be closely monitored. Thus, there is a need for more resources to be invested to promote quality antenatal care information dissemination and service provision via web-based platforms.

The findings of this study should be interpreted with caution. This is a web-based survey, wherein participants were self-selected, tended to have received higher education, possessed digital devices, and were possibly more health conscious, all of which might introduce selection bias and not be representative of all pregnant women. The results may also be subjected to recall bias, as the variables are based on self-reporting. The cross-sectional design of this study prevents significant causal associations to be demonstrated. Finally, the residence location of participants was not geographically evenly distributed; only a small proportion of pregnant women resided in Hubei Province—the region most severely affected area by COVID-19 in China.

### Conclusions

In the early stage of the COVID-19 epidemic in China, a large proportion of pregnant women experienced high levels of perceived stress, anxiety, and depression. In addition, a significantly lower risk of maternal mental health disorders was found to be associated with accessing professional antenatal care information via hospitals’ WOAs. These findings advocate for antenatal health service providers to develop evidence-based health care information and update service provision information on social media platforms in a timely manner. This should also form an essential component of public health preparedness and response to mitigate negative mental health outcomes among pregnant women.

## Appendix

Multimedia Appendix 1 Questions about COVID-19 self-protection behaviors and antenatal care knowledge included in the survey questionnaire.

Multimedia Appendix 2 Mental health disorders among Chinese pregnant women accessing antenatal care information via hospitals’ official accounts on social media platforms during the COVID-19 outbreak (N=1873).

Multimedia Appendix 3 Mental health disorders among Chinese pregnant women accessing antenatal care information via hospitals’ hotlines or SMS during the COVID-19 outbreak (N=1873).

Multimedia Appendix 4 Mental health disorders among Chinese pregnant women accessing antenatal care information via friends or family members during the COVID-19 outbreak (N=1873).

## Abbreviations

CPSS: Chinese Perceived Stress Scale
EDS: Edinburgh Depression Scale
MCH: maternal and child health
OR: odds ratio
QR: quick response
SAS: Self-Rating Anxiety Scale (SAS)
WOA: WeChat or Weibo official account
