# Supplementary material for: Maternal Mental Health Status and Approaches for Accessing Antenatal Care Information During the COVID-19 Epidemic in China: Cross-Sectional Study
Source: J Med Internet Res. 2021 Jan 18;23(1):e18722. doi: 10.2196/18722 (PMC7817253; doi:10.2196/18722)
Supplement: Multimedia Appendix 1 [file jmir_v23i1e18722_app1.docx]

**Supplementary material - Questions on COVID-19 self-protection behaviors and antenatal care knowledge in the questionnaire used for the survey**

**COVID-19 self-protection behaviors in the questionnaire**

1. Have you tried to avoid going out and staying at home in the past seven days?
2. Yes (skip to no.2) (2) No. If NO, what were the reasons:________
3. **How often do you wear a facemask when going out?**
4. Always (2) Often (3) Sometimes (4) Seldom (5) Never (6) Not applicable (Never go outside)
5. **Which of the following statement describes what you have been doing since the Covid-19 started? (Multiple choice)**
6. Open windows and ventilate the room regularly.
7. Take body temperature when feel feverish.
8. Avoid using public transport as much as possible.
9. Avoid going to crowded places.
10. Avoid contacting with/buying/eating wild animals.
11. Tell your doctor or community health worker about your travel history in COVID-19 epidemic area.
12. **Which of the following statement describes your self-protection practice? (Multiple choice)**
13. Cover your mouth when cough or sneeze with a tissue or elbow.
14. Avoid touching your mouth, eyes and nose with hands.
15. Wash hands with hand sanitizer or soap before eating and after defecation, especially after returning from outside.
16. Avoid touching door knob, lift button, hand railing in public places directly with your hands.

**COVID-19 related antenatal care knowledge in the questionnaire**

1. **How do you think about the delay or cancelation of the antenatal care visit?**
2. It is appropriate to delay or cancel all antenatal care visit.
3. Pregnant women in the first trimester do not need to have antenatal care visit.
4. Pregnant women in the second trimester do not need to have antenatal care visit.
5. Pregnant women in the third trimester do not need to have antenatal examinations.
6. **Can a pregnant woman with suspected COVID-19 infection undergo CT examination?**
7. No.
8. Yes, CT can be used for chest examination when the abdomen is fully protected.
9. **Under which following circumstances should a pregnant woman go to hospital (select correct statement)? (Multiple choice)**
10. Coming from COVID-19 epidemic areas or close contact with confirmed COVID-19 cases within 14 days, oral temperature>37.3℃ and with coughing, fatigue, symptoms, the pregnant woman should go to hospital.
11. Coming from COVID-19 epidemic areas or close contact with confirmed COVID-19 cases within 14 days, with normal temperature but is short of breath or feels difficult to breath, the pregnant woman should go to hospital.
12. Not coming from COVID-19 epidemic areas or without the contact history with COVID-19 confirmed cases, but with the oral temperature>38℃, the pregnant woman should go to hospital.
13. Not coming from COVID-19 epidemic areas or without the contact history with COVID-19 confirmed cases, with the oral temperature>38℃, but if it is considered to be a common cold, the pregnant woman only needs to rest at home instead of going to hospital.
14. Experiencing obstetric symptoms (uterine contractions, hemorrhage or other symptoms) which require timely medical attention, the pregnant woman should go to hospital.
15. I don’t know.
16. **Where should a pregnant woman go if she has fever? (Multiple choice)**
17. Directly go to a fever clinic.
18. Directly go to a maternity hospital.
19. If she has simple respiratory symptoms or fever but no obstetrical symptoms (uterine contractions, hemorrhage), go to a fever clinic in designated hospitals for treating COVID-19 which has an obstetric department.
20. If she is also experiencing obstetric symptoms, go to the designated hospitals for treating COVID-19, without checking whether having obstetric department.
21. I don’t know.
22. **Should a neonate be quarantined if the mother is suspected of or confirmed with COVID-19? (Multiple choice)**
23. If the mother is confirmed with COVID-19, the neonate should be quarantined and monitored for adverse health conditions.
24. If the mother is suspected of or confirmed with COVID-19, the neonate should be quarantined and observed for 14 days.
25. If the mother is suspected of or confirmed with COVID-19, the neonate can stay in the same room with mother.
26. If the newborn is suspected or confirmed with COVID-19, the neonate should be separated from the mother and transferred to a different room.
27. I don’t know.
28. **Can women with confirmed COVID-19 continue to breastfeed?**
29. No, Breastfeeding is not recommended.
30. Yes, Breastfeeding is encouraged.
31. I don’t know.
