# Supplementary material for: Maternal Mental Health Status and Approaches for Accessing Antenatal Care Information During the COVID-19 Epidemic in China: Cross-Sectional Study
Source: J Med Internet Res. 2021 Jan 18;23(1):e18722. doi: 10.2196/18722 (PMC7817253; doi:10.2196/18722)
Supplement: Multimedia Appendix 2 [file jmir_v23i1e18722_app2.docx]

**Supplementary table 1 Accessing antenatal care information via hospitals’ official accounts on social media platforms and mental health disorders among Chinese pregnant women during COVID-19 epidemic ^a^ (n=1,873)**

|  | **Perceived stress** | | | **Anxiety** | |  | **Depression** | |  |
| --- | --- | --- | --- | --- | --- | --- | --- | --- | --- |
|  | **cOR** | **aOR(95% CI)** | **P** | **cOR** | **aOR(95% CI)** | **P** | **cOR** | **aOR95% CI** | **P** |
| **Age** |  |  |  |  |  |  |  |  |  |
| <29 | 1.00 | 1.00 | / | 1.00 | 1.00 | / | 1.00 | 1.00 | / |
| ≥29 | 0.78(0.58,1.06) | 0.92(0.66,1.28) | .62 | 0.87(0.68,1.10) | 0.96(0.73,1.24) | .73 | 0.96(0.80,1.16) | 1.02(0.83,1.25) | .87 |
| **Education** |  |  |  |  |  |  |  |  |  |
| ≤Junior-high | 1.00 | 1.00 | / | 1.00 | 1.00 | / | 1.00 | 1.00 | / |
| Senior high | 0.57(0.26,1.24) | 0.67(0.31,1.48) | .32 | 0.72(0.48,1.08) | 0.77(0.51,1.17) | .22 | 1.03(0.72,1.47) | 1.12(0.78,1.61) | .54 |
| ≥College | 0.32(0.17,0.62) | 0.45(0.23,0.92) | .03 | 0.45(0.32,0.61) | 0.52(0.36,0.77) | .001 | 0.62(0.47,0.83) | 0.75(0.54,1.04) | .08 |
| **Employment status** | |  |  |  |  |  |  |  |  |
| Unemployed | 1.00 | 1.00 | / | 1.00 | 1.00 | / | 1.00 | 1.00 | / |
| Employed | 0.59(0.36,0.98) | 0.81(0.48,1.38) | .44 | 0.78(0.56,1.08) | 1.03(0.72,1.47) | .87 | 0.79(0.60,1.03) | 0.93(0.70,1.24) | .63 |
| **Parity** |  |  |  |  |  |  |  |  |  |
| Primiparous | 1.00 | 1.00 | / | 1.00 | 1.00 | / | 1.00 | 1.00 | / |
| Multiparous | 1.13(0.83,1.54) | 0.96(0.68,1.35) | .81 | 1.21(0.95,1.54) | 1.04(0.79,1.36) | .80 | 1.26(1.04,1.53) | 1.13(0.91,1.39) | .26 |
| **Trimester** |  |  |  |  |  |  |  |  |  |
| 1^st^ | 1.00 | 1.00 | / | 1.00 | 1.00 | / | 1.00 | 1.00 | / |
| 2^nd^ | 1.18(0.84,1.66) | 0.92(0.65,1.31) | .65 | 0.93(0.70,1.24) | 0.74(0.54,1.00) | .052 | 1.05(0.84,1.30) | 0.92(0.73,1.15) | .45 |
| 3^rd^ | 1.38(0.95,1.99) | 0.95(0.63,1.43) | .80 | 1.09(0.81,1.47) | 0.80(0.57,1.12) | .20 | 1.09(0.86,1.37) | 0.92(0.71,1.19) | .53 |
| **Living area** |  |  |  |  |  |  |  |  |  |
| Urban | 1.00 | 1.00 | / | 1.00 | 1.00 | / | 1.00 | 1.00 | / |
| Suburban | 1.04(0.71,1.51) | 0.76(0.37,1.53) | .44 | 1.02(0.75,1.41) | 1.01(0.67,1.51) | .98 | 1.09(0.86,1.39) | 0.84(0.60,1.17) | .29 |
| Rural | 2.68(1.39,5.16) | 0.65(0.31,1.38) | .26 | 1.63(1.16,2.30) | 0.86(0.55,1.36) | .52 | 1.59(1.19,2.13) | 0.82(0.57,1.19) | .31 |
| **Current residence** | | 1.00 |  |  |  |  |  |  |  |
| Non-Shanghai | 1.00 | 1.00 | / | 1.00 | 1.00 | / | 1.00 | 1.00 | / |
| Shanghai | 0.42(0.27,0.64) | 0.48(0.30,0.77) | .002 | 0.69(0.53,0.90) | 0.73(0.54,1.00) | .047 | 0.78(0.64,0.97) | 0.85(0.67,1.09) | .20 |
| **Pregnancy complications** | |  |  |  |  |  |  |  |  |
| No | 1.00 | 1.00 | / | 1.00 | 1.00 | / | 1.00 | 1.00 | / |
| Yes | 1.32(0.93,1.88) | 1.22(0.85,1.77) | .28 | 1.35(1.05,1.75) | 1.35(1.03,1.77) | .03 | 1.15(0.94,1.42) | 1.12(0.90,1.39) | .32 |
| **Score of COVID-19 prevention self-protection behaviors** | | | | | | | | | |
| Low | 1.00 | 1.00 | / | 1.00 | 1.00 | / | 1.00 | 1.00 | / |
| High | 0.83(0.60,1.15) | 1.00(0.71,1.40) | .99 | 0.90(0.68,1.19) | 1.05(0.78,1.40) | .76 | 0.95(0.76,1.17) | 1.05(0.84,1.30) | .69 |
| **Score of COVID-19 antenatal care knowledge** | | | | | | | | | |
| Low | 1.00 | 1.00 | / | 1.00 | 1.00 | / | 1.00 | 1.00 | / |
| High | 0.60(0.44,0.81) | 0.64(0.47,0.87) | .005 | 0.67(0.50,0.90) | 0.74(0.55,0.99) | .04 | 0.81(0.66,1.00) | 0.85(0.69,1.06) | .15 |
| **Access to antenatal care information via hospitals’ official accounts on social media platforms** | | | | | | | | | |
| No | 1.00 | 1.00 | / | 1.00 | 1.00 | / | 1.00 | 1.00 | / |
| Yes | 0.40(0.26,0.61) | 0.46(0.30,0.72) | .001 | 0.48(0.37,0.62) | 0.53(0.41,0.68) | <.001 | 0.67(0.54,0.83) | 0.73(0.59,0.91) | .005 |

^a^Multiple binary logistic regression
