# Supplementary material for: Maternal Mental Health Status and Approaches for Accessing Antenatal Care Information During the COVID-19 Epidemic in China: Cross-Sectional Study
Source: J Med Internet Res. 2021 Jan 18;23(1):e18722. doi: 10.2196/18722 (PMC7817253; doi:10.2196/18722)
Supplement: Multimedia Appendix 3 [file jmir_v23i1e18722_app3.docx]

**Supplementary table 2 Accessing antenatal care information via hospitals’ hotlines or SMS and mental health disorders among Chinese pregnant women during COVID-19 epidemic ^a^ (n=1,873)**

|  | **Perceived stress** | | | **Anxiety** | |  | **Depression** | |  |
| --- | --- | --- | --- | --- | --- | --- | --- | --- | --- |
|  | **cOR** | **aOR(95% CI)** | **P** | **cOR** | **aOR(95% CI)** | **P** | **cOR** | **aOR95% CI** | **P** |
| **Age** |  |  |  |  |  |  |  |  |  |
| <29 | 1.00 | 1.00 | / | 1.00 | 1.00 | / | 1.00 | 1.00 | / |
| ≥29 | 0.78(0.58,1.06) | 0.91(0.65,1.26) | .56 | 0.87(0.68,1.10) | 0.94(0.72,1.22) | .63 | 0.96(0.80,1.16) | 1.01(0.82,1.24) | .93 |
| **Education** |  |  |  |  |  |  |  |  |  |
| ≤Junior-high | 1.00 | 1.00 | / | 1.00 | 1.00 | / | 1.00 | 1.00 | / |
| Senior high | 0.57(0.26,1.24) | 0.66(0.30,1.44) | .28 | 0.72(0.48,1.08) | 0.75(0.50,1.13) | .17 | 1.03(0.72,1.47) | 1.11(0.77,1.59) | .59 |
| ≥College | 0.32(0.17,0.62) | 0.41(0.21,0.83) | .01 | 0.45(0.32,0.61) | 0.47(0.32,0.68) | <.001 | 0.62(0.47,0.82) | 0.71(0.52,0.98) | .04 |
| **Employment status** | |  |  |  |  |  |  |  |  |
| Unemployed | 1.00 | 1.00 | / | 1.00 | 1.00 | / | 1.00 | 1.00 | / |
| Employed | 0.59(0.36,0.98) | 0.82(0.49,1.39) | .46 | 0.78(0.56,1.08) | 1.02(0.72,1.45) | .93 | 0.79(0.60,1.03) | 0.93(0.70,1.23) | .61 |
| **Parity** |  |  |  |  |  |  |  |  |  |
| Primiparous | 1.00 | 1.00 | / | 1.00 | 1.00 | / | 1.00 | 1.00 | / |
| Multiparous | 1.13(0.83,1.54) | 0.97(0.69,1.37) | .88 | 1.21(0.95,1.54) | 1.04(0.79,1.37) | .76 | 1.26(1.04,1.53) | 1.13(0.92,1.40) | .25 |
| **Trimester** |  |  |  |  |  |  |  |  |  |
| 1^st^ | 1.00 | 1.00 | / | 1.00 | 1.00 | / | 1.00 | 1.00 | / |
| 2^nd^ | 1.18(0.84,1.66) | 0.95(0.67,1.36) | .79 | 0.93(0.70,1.24) | 0.77(0.57,1.04) | .09 | 1.05(0.84,1.30) | 0.93(0.74,1.17) | .54 |
| 3^rd^ | 1.38(0.95,1.99) | 0.94(0.63,1.42) | .77 | 1.09(0.81,1.47) | 0.80(0.57,1.13) | .20 | 1.09(0.86,1.37) | 0.92(0.71,1.19) | .53 |
| **Living area** |  |  |  |  |  |  |  |  |  |
| Urban | 1.00 | 1.00 | / | 1.00 | 1.00 | / | 1.00 | 1.00 | / |
| Suburban | 1.04(0.71,1.51) | 0.71(0.35,1.43) | .33 | 1.02(0.75,1.41) | 0.93(0.62,1.38) | .70 | 1.09(0.86,1.39) | 0.81(0.58,1.12) | .27 |
| Rural | 2.68(1.39,5.16) | 0.60(0.29,1.28) | .19 | 1.63(1.16,2.30) | 0.80(0.51,1.25) | .33 | 1.59(1.19,2.13) | 0.80(0.55,1.15) | .29 |
| **Current residence** | | 1.00 |  |  |  |  |  |  |  |
| Non-Shanghai | 1.00 | 1.00 | / | 1.00 | 1.00 | / | 1.00 | 1.00 | / |
| Shanghai | 0.42(0.27,0.64) | 0.49(0.31,0.79) | .003 | 0.69(0.53,0.90) | 0.75(0.55,1.03) | .07 | 0.78(0.64,0.97) | 0.86(0.68,1.10) | .23 |
| **Pregnancy complications** | |  |  |  |  |  |  |  |  |
| No | 1.00 | 1.00 | / | 1.00 | 1.00 | / | 1.00 | 1.00 | / |
| Yes | 1.32(0.93,1.88) | 1.25(0.87,1.80) | .24 | 1.35(1.05,1.75) | 1.36(1.04,1.79) | .03 | 1.15(0.94,1.42) | 1.12(0.91,1.40) | .29 |
| **Score of COVID-19 prevention self-protection behaviors** | | | | | | | | | |
| Low | 1.00 | 1.00 | / | 1.00 | 1.00 | / | 1.00 | 1.00 | / |
| High | 0.83(0.60,1.15) | 1.01(0.72,1.42) | .95 | 0.90(0.68,1.19) | 1.06(0.79,1.43) | .68 | 0.95(0.76,1.17) | 1.05(0.84,1.31) | .66 |
| **Score of COVID-19 antenatal care knowledge** | | | | | | | | | |
| Low | 1.00 | 1.00 | / | 1.00 | 1.00 | / | 1.00 | 1.00 | / |
| High | 0.60(0.44,0.81) | 0.62(0.45,0.85) | .003 | 0.67(0.50,0.90) | 0.71(0.53,0.95) | .02 | 0.81(0.66,1.00) | 0.84(0.68,1.04) | .11 |
| **Access to antenatal care information via hospitals’ hotlines or SMS^b^** | | | | | | | | | |
| No | 1.00 | 1.00 | / | 1.00 | 1.00 | / | 1.00 | 1.00 | / |
| Yes | 0.86(0.64,1.14) | 0.86(0.64,1.16) | .32 | 0.83(0.65,1.05) | 0.77(0.60,0.98) | .04 | 0.95(0.79,1.14) | 0.91(0.76,1.10) | .35 |

^a^Multiple binary logistic regression; ^b^Short message service
