# Supplementary material for: Maternal Mental Health Status and Approaches for Accessing Antenatal Care Information During the COVID-19 Epidemic in China: Cross-Sectional Study
Source: J Med Internet Res. 2021 Jan 18;23(1):e18722. doi: 10.2196/18722 (PMC7817253; doi:10.2196/18722)
Supplement: Multimedia Appendix 4 [file jmir_v23i1e18722_app4.docx]

**Supplementary table 3 Accessing antenatal care information from friends or family members and mental health disorders among Chinese pregnant women during COVID-19 epidemic ^a^ (n=1,873)**

|  | **Perceived stress** | | | | **Anxiety** | |  | **Depression** | |  |
| --- | --- | --- | --- | --- | --- | --- | --- | --- | --- | --- |
|  | **cOR** | **aOR(95% CI)** | | **P** | **cOR** | **aOR(95% CI)** | **P** | **cOR** | **aOR95% CI** | **P** |
| **Age** |  |  | |  |  |  |  |  |  |  |
| <29 | 1.00 | 1.00 | | / | 1.00 | 1.00 | / | 1.00 | 1.00 | / |
| ≥29 | 0.78(0.58,1.06) | 0.90(0.65,1.26) | | .55 | 0.87(0.68,1.10) | 0.94(0.73,1.23) | .67 | 0.96(0.80,1.16) | 1.00(0.82,1.22) | .99 |
| **Education** |  |  | |  |  |  |  |  |  |  |
| ≤Junior-high | 1.00 | 1.00 | | / | 1.00 | 1.00 | / | 1.00 | 1.00 | / |
| Senior high | 0.57(0.26,1.24) | 0.67(0.30,1.46) | | .31 | 0.72(0.48,1.08) | 0.76(0.50,1.15) | .19 | 1.03(0.72,1.47) | 1.12(0.78,1.61) | .53 |
| ≥College | 0.32(0.17,0.62) | 0.42(0.21,0.83) | | .01 | 0.45(0.32,0.61) | 0.48(0.33,0.70) | <.001 | 0.62(0.47,0.83) | 0.72(0.52,0.99) | .04 |
| **Employment status** | | |  |  |  |  |  |  |  |  |
| Unemployed | 1.00 | 1.00 | | / | 1.00 | 1.00 | / | 1.00 | 1.00 | / |
| Employed | 0.59(0.36,0.98) | 0.81(0.48,1.38) | | .44 | 0.78(0.56,1.08) | 1.01(0.71,1.43) | .97 | 0.79(0.60,1.03) | 0.92(0.69,1.22) | .56 |
| **Parity** |  |  | |  |  |  |  |  |  |  |
| Primiparous | 1.00 | 1.00 | | / | 1.00 | 1.00 | / | 1.00 | 1.00 | / |
| Multiparous | 1.13(0.83,1.54) | 0.97(0.69,1.36) | | .86 | 1.21(0.95,1.54) | 1.02(0.78,1.34) | .89 | 1.26(1.04,1.53) | 1.14(0.92,1.40) | .24 |
| **Trimester** |  |  | |  |  |  |  |  |  |  |
| 1^st^ | 1.00 | 1.00 | | / | 1.00 | 1.00 | / | 1.00 | 1.00 | / |
| 2^nd^ | 1.18(0.84,1.66) | 0.97(0.68,1.38) | | .86 | 0.93(0.70,1.24) | 0.78(0.58,1.06) | .12 | 1.05(0.84,1.30) | 0.94(0.75,1.19) | .62 |
| 3^rd^ | 1.38(0.95,1.99) | 0.96(0.64,1.44) | | .84 | 1.09(0.81,1.47) | 0.82(0.59,1.15) | .26 | 1.09(0.86,1.37) | 0.93(0.72,1.20) | .57 |
| **Living area** |  |  | |  |  |  |  |  |  |  |
| Urban | 1.00 | 1.00 | | / | 1.00 | 1.00 | / | 1.00 | 1.00 | / |
| Suburban | 1.04(0.71,1.51) | 0.70(0.35,1.41) | | .32 | 1.02(0.75,1.41) | 0.92(0.62,1.38) | .70 | 1.09(0.86,1.39) | 0.80(0.57,1.11) | .18 |
| Rural | 2.68(1.39,5.16) | 0.60(0.28,1.28) | | .19 | 1.63(1.16,2.30) | 0.80(0.51,1.26) | .34 | 1.59(1.19,2.13) | 0.79(0.55,1.14) | .21 |
| **Current residence** | | |  |  |  |  |  |  |  |  |
| Non-Shanghai | 1.00 | 1.00 | | / | 1.00 | 1.00 | / | 1.00 | 1.00 | / |
| Shanghai | 0.42(0.27,0.64) | 0.49(0.31,0.78) | | .003 | 0.69(0.53,0.90) | 0.74(0.54,1.00) | .051 | 0.78(0.64,0.97) | 0.86(0.67,1.10) | .22 |
| **Pregnancy complications** | | |  |  |  |  |  |  |  |  |
| No | 1.00 | 1.00 | | / | 1.00 | 1.00 | / | 1.00 | 1.00 | / |
| Yes | 1.32(0.93,1.88) | 1.25(0.86,1.80) | | .24 | 1.35(1.05,1.75) | 1.34(1.02,1.76) | .03 | 1.15(0.94,1.42) | 1.12(0.90,1.39) | .30 |
| **Score of COVID-19 prevention self-protection behaviors** | | | | | | | | | | |
| Low | 1.00 | 1.00 | | / | 1.00 | 1.00 | / | 1.00 | 1.00 | / |
| High | 0.83(0.60,1.15) | 0.98(0.70,1.38) | | .90 | 0.90(0.68,1.19) | 1.03(0.77,1.38) | .84 | 0.95(0.76,1.17) | 1.02(0.82,1.28) | .85 |
| **Score of COVID-19 antenatal care knowledge** | | | | | | | | | | |
| Low | 1.00 | 1.00 | | / | 1.00 | 1.00 | / | 1.00 | 1.00 | / |
| High | 0.60(0.44,0.81) | 0.62(0.45,0.84) | | .003 | 0.67(0.50,0.90) | 0.71(0.53,0.96) | .03 | 0.81(0.66,1.00) | 0.83(0.67,1.03) | .10 |
| **Access to antenatal care information from friends or family members** | | | | | | | | | | |
| No | 1.00 | 1.00 | |  | 1.00 | 1.00 |  | 1.00 | 1.00 | / |
| Yes | 1.25(0.93,1.70) | 1.34(0.98,1.82) | | .07 | 1.03(0.81,1.31) | 1.08(0.84,1.37) | .56 | 1.26(1.05,1.52) | 1.32(1.09,1.60) | .004 |

^a^Multiple binary logistic regression
